# Multi-Scale Tolerance Mechanisms of *Xanthium strumarium* L. Under Lead Stress and Its Application in Phytoremediation

**DOI:** 10.3390/plants14091307

**Published:** 2025-04-26

**Authors:** Shilin Xu, Xiaofang Wang, Zichen Meng, Pingyao Cheng, Wei Li, You Zhou, Yongsheng Li

**Affiliations:** College of Forestry, Henan Agricultural University, Zhengzhou 450046, China; smallx_beat@outlook.com (S.X.); xiaofangwang1868@163.com (X.W.); 13730101363@163.com (Z.M.); 13920842141@163.com (P.C.); 15236900487@163.com (W.L.); zhouyou7521@163.com (Y.Z.)

**Keywords:** lead pollution, phytoremediation, *Xanthium strumarium* L., heavy metal tolerance mechanisms

## Abstract

Heavy metal pollution poses a global environmental challenge, with lead (Pb) being particularly concerning due to its persistence and toxicity. This study investigated *Xanthium strumarium* L. from China’s Yellow River Sanmenxia section through hydroponic experiments (0–600 mg/L Pb^2+^, 1–11 d exposure) to elucidate its Pb^2+^ response mechanisms. Integrated analyses (EDX, FTIR, thermogravimetry, hyperspectral imaging) revealed a three-phase sequestration strategy: the roots immobilized 88.55% of Pb through pectin carboxyl de-esterification and lignin–Pb complexation, while the stems and leaves retained <11.14% and <0.31%, respectively. A critical threshold (300 mg/L) triggered nonlinear Pb accumulation escalation. Thermogravimetric analysis demonstrated enhanced cell wall stability under Pb stress (66.7% residual carbon increase at 600 mg/L). Hyperspectral features (1670 nm band intensity) effectively tracked physiological stress dynamics. The findings establish *X. strumarium*’s superior suitability for root-based immobilization rather than phytoextraction in Pb-contaminated sites, with its low translocation efficiency minimizing ecological risks. The identified concentration threshold and spectral biomarkers provide multi-scale insights for optimizing in situ phytostabilization strategies, advancing both theoretical understandings and practical applications in heavy metal remediation.

## 1. Introduction

Lead (Pb) pollution remains a global environmental challenge due to its persistent nature and significant ecological risks. Natural soil Pb concentrations in certain sections of the Yellow River Basin reach approximately 75 mg/kg [1,2], representing 1–2 times the regional background levels [3,4] and slightly exceeding China’s agricultural soil risk screening threshold (70 mg/kg, GB 15618-2018) [5]. More concerningly, heavily industrialized zones in the Pearl River and Yangtze River Basins exhibit substantially elevated Pb concentrations of 189 mg/kg and 413 mg/kg, respectively [6]. The primary contamination sources include mining operations, historical leaded gasoline residues, landfill emissions, and electronic waste [7,8,9,10]. Contaminants migrate through colloidal adsorption into soils or via runoff into aquatic systems [11,12], subsequently accumulating through trophic transfer with documented impacts on human neurological, renal, and hematopoietic functions [13]. Distinct from other polluted areas, this region’s dynamic hydrogeochemical processes—particularly flood-induced redox cycling and colloidal transport—fundamentally modify heavy metal bioavailability [14,15]. These unique environmental parameters establish an ideal natural laboratory for investigating plant adaptation mechanisms under fluctuating metal stress conditions. The remediation strategies developed for this fluvial system hold substantial reference value for managing contamination in global alluvial ecosystems with analogous sedimentary and hydrological characteristics.

Among heavy metal remediation approaches, physical–chemical techniques such as soil leaching and solidification/stabilization demonstrate rapid pollution reduction capabilities, yet face constraints in large-scale implementation due to their elevated costs, secondary contamination risks, and ecosystem disruption [16,17]. Phytoremediation has emerged as a prominent research focus for sustainable management strategies owing to its environmentally benign nature and economic viability [18]. Nevertheless, conventional hyperaccumulator species frequently encounter limitations, including restricted biomass production and poor regional acclimatization [19]. *Xanthium strumarium* L., a species widely distributed from pantropical to temperate regions, demonstrates notable ecological advantages, encompassing robust adaptability, extensive geographical range, rapid growth rates, and low palatability to herbivores [20,21]. Seed lineages subjected to prolonged oxidative stress selection pressures during the Yellow River’s historical flood cycles may have developed distinctive metal tolerance phenotypes compared to conventional populations. Existing research confirms *X. strumarium*’s significant tolerance to zinc and cadmium contamination [22,23], while its physiological adaptation mechanisms and remediation efficacy under Pb stress remain to be systematically elucidated.

The tolerance mechanisms of plants to heavy metals involve multifaceted physiological and biochemical responses, including cell wall sequestration, vacuolar compartmentalization, the synthesis of metal-chelating proteins, and the activation of antioxidant defense systems [24,25]. Recent studies have proposed divergent hypotheses regarding the underlying mechanisms of heavy metal tolerance: one suggests the passive accumulation of metals in roots via the extracellular matrix pathway [26], while others propose that active transport proteins (e.g., the HMA family) mediate metal translocation to aerial tissues [27,28]. This discrepancy underscores the complexity of plant–metal interactions, with current research often limited to static experimental designs (single time points or concentrations) which hinder the elucidation of dynamic adaptive processes. Traditional physiological assays, such as analyses of malondialdehyde (MDA) content and superoxide dismutase (SOD) activity [29,30], provide insights into oxidative stress but fail to decode molecular mechanisms, such as changes in cell wall composition or metal coordination morphology.

Although plant responses to heavy metal stress have garnered considerable attention, current research remains constrained by critical limitations. Static experimental designs fail to capture dynamic adaptive processes, while conventional physiological indicators inadequately resolve molecular mechanisms such as cell wall remodeling and metal coordination speciation. Furthermore, controlled cultivation in artificial climate chambers, while minimizing environmental variability, may distort tolerance assessments compared to natural populations, compromising ecological relevance. This study addresses these knowledge gaps through multi-tiered time–concentration gradient experiments conducted on wild populations to preserve ecological authenticity, specifically elucidating the Pb immobilization mechanisms dominated by *X. strumarium*’s root systems and their cross-tissue regulatory networks. Moving beyond traditional single-scale analyses, we integrate multidimensional evidence encompassing Pb spatiotemporal distribution dynamics, the functional reorganization of cell wall components, and physiological stabilization patterns. Our findings unveil the ecological adaptation strategy of *X. strumarium*’s rhizosphere sequestration mechanism, establishing a novel cross-scale analytical framework that bridges molecular mechanisms with ecosystem-level metal tolerance. This work advances the targeted screening of hyper-adaptive phytoremediation resources and provides critical insights for optimizing in situ phytostabilization technologies, ultimately facilitating the integration of molecular discoveries into practical ecological restoration applications.

## 2. Results

### 2.1. Pb Spatiotemporal Distribution

**Figure 1:**
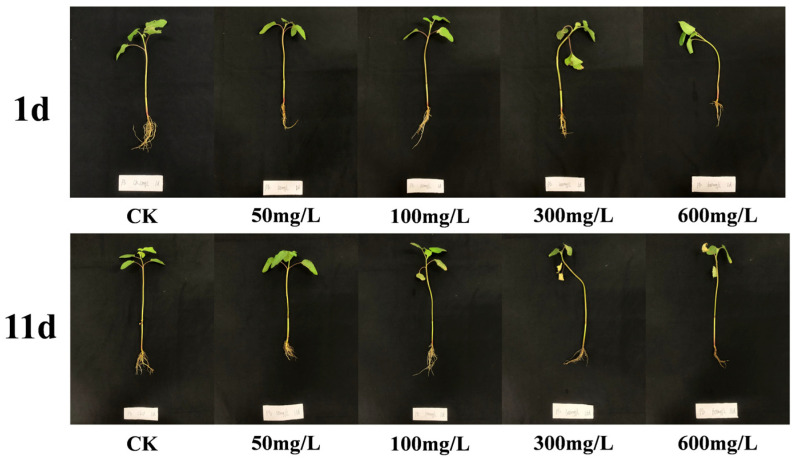
Changes in Pb concentration in *X. strumarium* at 1 and 11 days of cultivation.

The phenotypic responses of *X. strumarium* to Pb exposure are shown in Figure 1. After 1-day treatment, the stem and branch tissues exhibited pronounced wilting symptoms at 300 mg/L and 600 mg/L Pb^2+^ concentrations. Compared to CK, the 600 mg/L treatment group displayed significant root biomass reduction, indicating acute growth inhibition. Following 11-day exposure, progressive chlorosis developed in foliar tissues with increasing Pb^2+^ concentrations, accompanied by further root system deterioration. Severe phytotoxicity manifested in the 300–600 mg/L groups through partial branch necrosis. These findings demonstrate the concentration-dependent and exposure-duration-dependent inhibitory effects of Pb^2+^ on *X. strumarium*’s morphological development.

**Figure 2:**
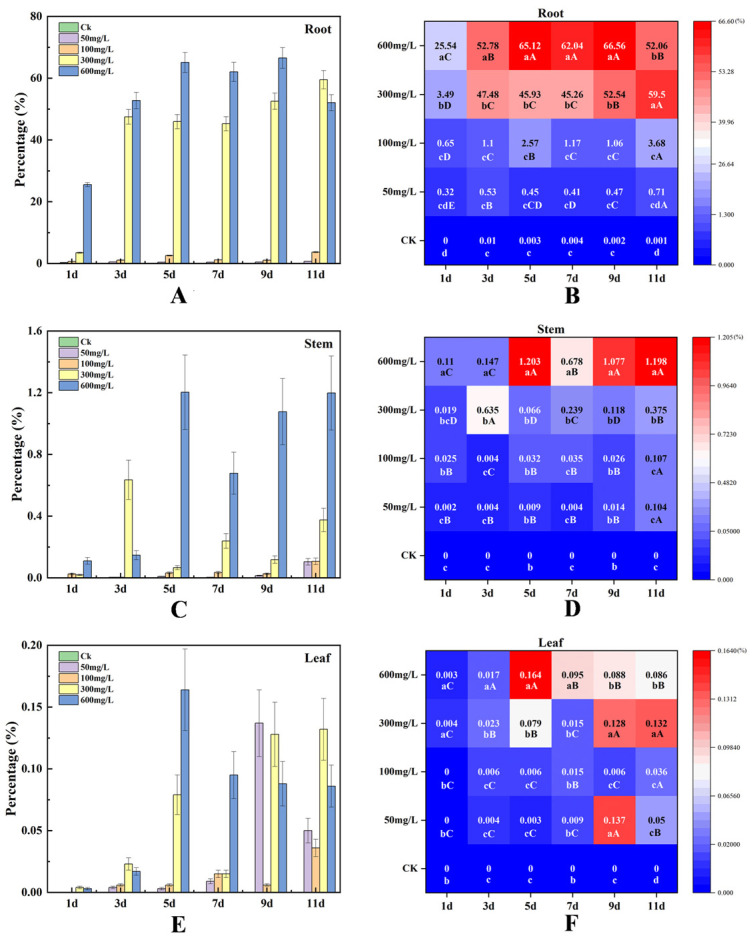
(**A**,**C**,**E**) show the relative Pb contents in the roots, stems, and leaves of *X. strumarium* under different treatments, respectively; (**B**,**D**,**F**) show the heatmaps of the roots, stems, and leaves of *X. strumarium* under different treatments, respectively. Lowercase letters indicate significant differences (*p* < 0.05) in relative Pb percentage content among Pb concentration levels at the same time point; uppercase letters denote significant differences (*p* < 0.05) across time points under identical Pb concentration levels.

The root systems served as the primary Pb accumulation sites, showing significant positive correlations (*p* < 0.001) between tissue Pb content and exposure concentration. The 600 mg/L treatment group exhibited the maximum accumulation efficiency, reaching 25.54% on Day 1 and peaking at 65.12% at Day 5—significantly exceeding lower concentration groups (Figure 2A). The low-concentration treatments (50–100 mg/L) demonstrated limited accumulation (<3.68%), while a nonlinear threshold effect emerged at 300 mg/L, triggering abrupt accumulation increases (>45%) (Figure 2B). Root (600 mg/L) Pb accumulation exhibited dynamic fluctuations along temporal and concentration gradients, reaching peak concentrations of 56,328.2 mg/kg in root tissues following 9-day exposure to maximum treatment levels.

Stem Pb^2+^ accumulation remained orders of magnitude lower than in the roots (<1.2%), yet displayed significant temporal escalation in the 600 mg/L group (0.11% at Day 1 to 1.20% at Day 11). Throughout the 11-day exposure, the stem Pb content in this group consistently exceeded that in the 50 mg/L treatment by 2.8–15.6× (Figure 2C). Notably, all concentration groups exhibited stem accumulation peaks during Days 5–9 (4–6 days post-root maxima), with the 600 mg/L group achieving 0.24%/day accumulation rates during this phase—7.3× higher than the low-concentration treatments (Figure 2D). ICP-OES analysis showed a peak Pb accumulation in stems (600 mg/L) of 7036.34 mg/kg on Day 5, decreasing to 6732.12 mg/kg by Day 9.

Leaf Pb^2+^ accumulation proved minimal (<0.16%), with no significant concentration-dependent increases observed, even at 600 mg/L (Figure 2E,F). The tissue-specific hierarchy in the 600 mg/L group was as follows: roots (25.54–66.56%) > stems (0.11–1.20%) > leaves (0.003–0.16%). Leaf (600 mg/L) tissues exhibited a maximum absolute Pb content of 233.82 mg/kg at Day 5.

Under short-term Pb^2+^ exposure (1–3 days), the 600 mg/L-treated roots demonstrated rapid accumulation escalation from 25.54% (Day 1) to 52.78% (Day 3), yielding a mean daily accumulation rate of 13.62%/d. During the mid-exposure phases (5–9 days), the root systems in 300–600 mg/L treatments maintained sustained accumulation (45–66%), while the stem (1.20%) and leaf (0.16%) peaks occurred at Day 5—exhibiting a 4-day phasic delay relative to root accumulation. Prolonged stress (11 days) induced notable root accumulation decline in the 600 mg/L group, decreasing 21.8% versus the Day 9 values (52.06% vs. 66.56%).

### 2.2. FTIR Characteristics

**Figure 3:**
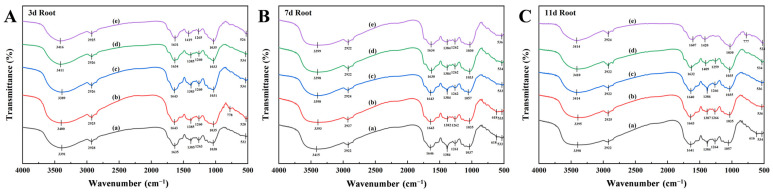
Infrared spectrograms of *X. strumarium* roots. (**A**) Infrared spectra after 3-day cultivation; (**B**) Infrared spectra after 7-day cultivation; (**C**) Infrared spectra after 11-day cultivation. (a) 0 mg/L; (b) 50 mg/L; (c) 100 mg/L; (d) 300 mg/L; (e) 600 mg/L.

FTIR spectral analysis revealed systematic peak variations in *X. strumarium* tissues under Pb stress (Figure 3). Characteristic absorption bands were identified across organs: broad O-H stretching vibrations (water/polysaccharide hydroxyl/phenolic -OH) at 3400–3390 cm^−1^ [31]; C-H stretching modes (aliphatic chains/lipids) at 2925 cm^−1^ [32]; amide I band (protein) overlapping aromatic C=C vibrations (lignin/phenolics) at 1630–1640 cm^−1^ [33,34]; COO^−^ symmetric stretching (pectin/uronic acids) coupled with C-H deformation (cellulose) at 1380–1420 cm^−1^ [35]; C-O stretching in cellulose/hemicellulose (1260–1265 cm^−1^) and aromatic ether linkages (lignin); polysaccharide C-O-C (1035 cm^−1^) and phospholipid C-O-P vibrations [36]; and distinct metal–oxygen (M-O) vibrations at 500–600 cm^−1^, indicative of Pb^2+^ coordination with carboxyl/phosphate groups [37].

The main infrared band characteristics of the roots are shown in Table 1. Short-term Pb^2+^ exposure (600 mg/L, Day 3) induced a 25 cm^−1^ upshift in O-H stretching vibrations (3416 cm^−1^ vs CK 3391 cm^−1^) within the 3400–3390 cm^−1^ band (Figure 3A), concurrent with spectral perturbation in the 1630–1640 cm^−1^ region. The mid-exposure phase (Day 7) revealed band convergence at 1380–1420 cm^−1^, suggesting enhanced pectin carboxyl (COO^−^) coordination. The lipid-associated 2925 cm^−1^ band maintained spectral stability (Δν < 3 cm^−1^) across all treatments, indicating preserved membrane integrity. Prolonged high-concentration stress (600 mg/L, Day 11) triggered a 34 cm^−1^ amide I band shift (1607 cm^−1^ vs CK 1641 cm^−1^), while moderate–low concentrations (50–100 mg/L) exhibited minimal peak displacement (Δν < 5 cm^−1^) throughout the experiment (Figure 3C).

**Figure 4:**
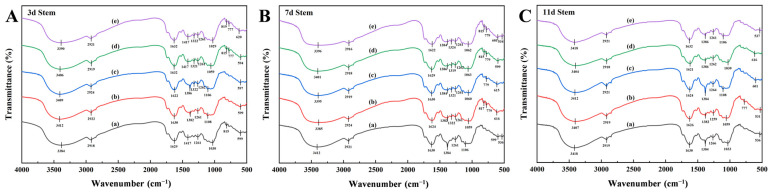
Infrared spectrograms of *X. strumarium* stems. (**A**) Infrared spectra after 3-day cultivation; (**B**) Infrared spectra after 7-day cultivation; (**C**) Infrared spectra after 11-day cultivation. (a) 0 mg/L; (b) 50 mg/L; (c) 100 mg/L; (d) 300 mg/L; (e) 600 mg/L.

The main infrared band characteristics of the stems are shown in Table 2. Short-term high-concentration exposure (600 mg/L, Day 3) induced the progressive intensification of the characteristic peaks at 3390 cm^−1^ (O-H stretching) and 1417 cm^−1^ (COO^−^ vibration) compared to CK (Figure 4A). The mid-exposure phase (Day 7) showed COO^−^ peak convergence to 1384–1386 cm^−1^ across treatments, with lipid-associated vibrations (2916–2924 cm^−1^) maintaining spectral stability (Figure 4B). Prolonged 600 mg/L stress (Day 11) caused the systematic alignment of spectral features toward the O-H stretching band at 3418 cm^−1^ (Figure 4C). The low-concentration groups (50–100 mg/L) exhibited sustained responses with 777 cm^−1^ (phytic acid P-O-C) and 1626 cm^−1^ (lignin C=C aromatic) vibrations, demonstrating spectral consistency (Δν < 3 cm^−1^).

**Figure 5:**
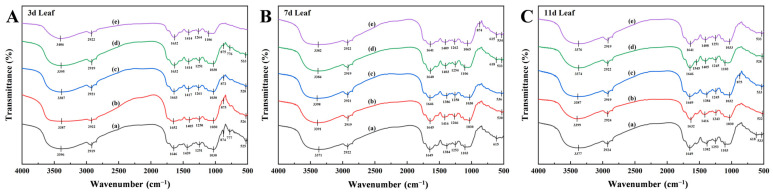
Infrared spectrograms of *X. strumarium* leaves. (**A**) Infrared spectra after 3-day cultivation; (**B**) Infrared spectra after 7-day cultivation; (**C**) Infrared spectra after 11-day cultivation. (a) 0 mg/L; (b) 50 mg/L; (c) 100 mg/L; (d) 300 mg/L; (e) 600 mg/L.

The main infrared band characteristics of the leaves are shown in Table 3. Short-term exposure (Day 3) revealed differential Pb^2+^ detoxification strategies: the 100 mg/L group showed phenolic–carboxyl coordination via 1652 cm^−1^ (phenolics) and 526 cm^−1^ (carboxylic acid) peaks, while the 600 mg/L group exhibited physical restriction mechanisms through 3406 cm^−1^ (polysaccharide O-H) and 1414 cm^−1^ (cell wall COO^−^) vibrations (Figure 5A). The mid-exposure phase (Day 7) demonstrated COO^−^ band convergence (1384–1416 cm^−1^) across treatments, coupled with organic acid signature intensification at 615 cm^−1^ (Figure 5B). Prolonged stress (Day 11) induced concentration-dependent responses: the low-concentration groups developed 522 cm^−1^ (metal-carboxylate) and 1632 cm^−1^ (amide I/lignin) vibrations, whereas the high-concentration treatments (600 mg/L) activated vacuolar sequestration signatures (533 cm^−1^, Pb–phosphate complexes) alongside metabolic suppression, evidenced by attenuated amide peaks (Figure 5C).

### 2.3. Thermogravimetric-Differential Scanning Calorimetry Analysis

**Figure 6:**
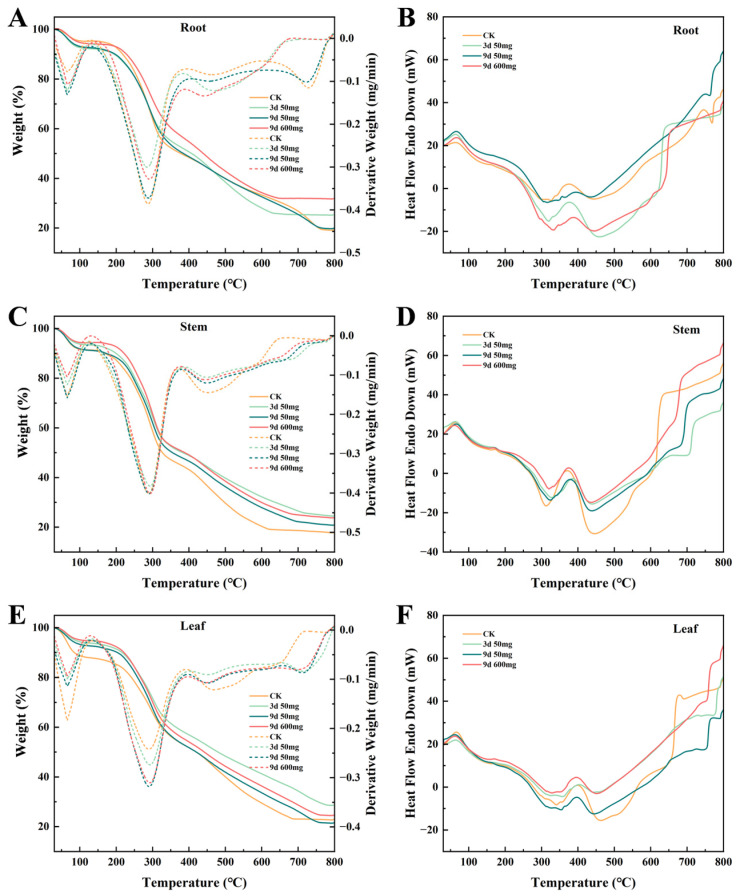
(**A**,**C**,**F**) show the TGA and DTG curves of the *X. strumarium* roots, stems, and leaves under four different treatments; (**B**,**D**,**F**) show the TSC curves of the roots, stems, and leaves under the same treatments.

Thermogravimetric analysis revealed Pb-induced changes in the pyrolysis characteristics of *X. strumarium* organs (Figure 6). During root dehydration (30–150 °C) (Figure 6A), the DTG curve showed a weight loss peak at 65 °C with a stronger intensity in the Pb-treated groups versus CK. CK exhibited higher residual mass at 129 °C. The DSC curves demonstrated broader CK endothermic peaks versus sharper Pb-treated group peaks, indicating altered moisture states from membrane damage (Figure 6B). In the 200–600 °C pyrolysis phase, CK showed greater main weight loss peak intensity at 288 °C. The DSC exothermic peak intensities (280–330 °C) were as follows: 9d/600 mg/L > 3d/50 mg/L > 9d/50 mg/L ≥ CK, with CK displaying broad peaks versus the treatment groups’ defined peaks. The residual carbon levels at 800 °C showed dose-dependence (Table 4): 9d/600 mg/L (31.855%, +66.7% vs. CK 19.121%), 3d/50 mg/L (25.242%), and 9d/50 mg/L (19.834%). This gradient aligns with the lignin–Pb composite accumulation trends.

The thermogravimetric analysis of the stems (Figure 6C) demonstrated concentration-dependent dehydration patterns during the low-temperature phase (30–150 °C), with the total weight loss being 8–9% across treatments. The CK group exhibited an 8.6% mass loss, while the 9d/600 mg/L treatment showed a significant reduction to 5.7% (42% decrease). Progressive Pb exposure induced linear weight loss decline, evidenced by the 22% lower dehydration in the 600 mg/L group versus the 50 mg/L groups. During medium-temperature pyrolysis (200–400 °C), CK specimens displayed a 17.7% mass loss at 289–370 °C, with Pb-treated groups showing differential responses (Table 5). The Corresponding DSC analysis (Figure 6D) revealed dual exothermic peaks at 280–350 °C and 400–460 °C where CK maintained maximum peak intensity versus the 9d/600 mg/L group’s attenuated broadened peaks. High-temperature carbonization analysis (450–800 °C) quantified residual the carbon content at 800 °C as 17.7% for CK, with the Pb^2+^-exposed specimens showing 35–50% increases (9d/600 mg/L: 23.7%).

The leaf thermogravimetric analysis revealed tissue-specific responses (Figure 6E). During low-temperature dehydration (30–150 °C), the CK group exhibited a 12.17% weight loss with distinct DTG peaks, while the Pb^2+^-treated groups showed reduced dehydration (9d/600 mg/L: 5.21%). The DSC curves demonstrated sharp CK endothermic peaks versus attenuated responses in the treated groups (Figure 6F). The mid-temperature pyrolysis (200–400 °C) diverged from the root–stem patterns: CK displayed minimal DTG peak intensity yet maintained higher cumulative weight loss than the treatment groups. The high-temperature carbonization (450–800 °C) residual carbon exhibited bi-phasic trends (Table 6): the 3d/50 mg/L group reached 28.65% (+25.8% vs. CK 22.78%), while the 9d/50 mg/L group showed a decline to 21.53% (−5.5% vs. CK).

### 2.4. Hyperspectral Principal Component Analysis

**Figure 7:**
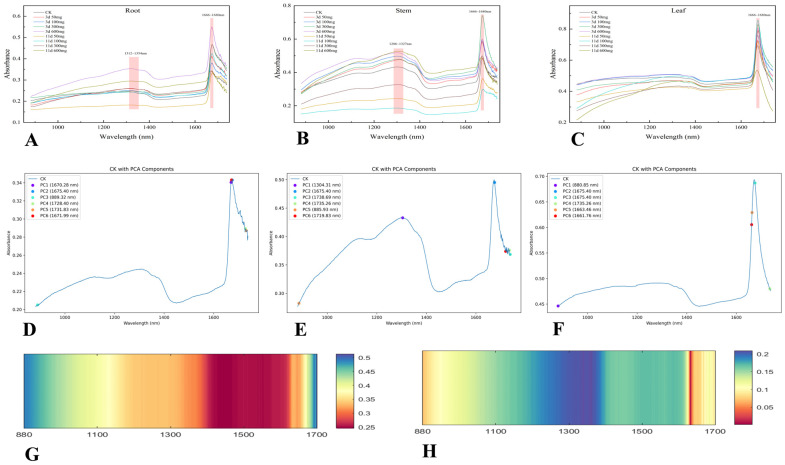
(**A**–**C**): spectral images of *X. strumarium* roots, stems, and leaves under different treatments; (**D**–**F**): PCA analysis of roots, stems, and leaves; (**G**,**H**): correlation analysis of spectral bands with time and concentration.

Figure 7A–C present the hyperspectral reflectance curves of the *X. strumarium* roots, stems, and leaves under two cultivation cycles (3 days and 11 days) and varying Pb stress concentrations (0, 50, 100, 300, 600 mg/L). All tissues exhibited significant reflection peaks in the 1666–1680 nm wavelength range, with the root and stem tissues additionally showing broad secondary peaks at 1312–1354 nm (as illustrated in Figure 7). To address the complexity of hyperspectral data, principal component analysis (PCA) was employed for dimensionality reduction. The analysis revealed that the first six principal components (PC1–PC6) were retained for the root, stem, and leaf tissues, collectively explaining the primary spectral variations observed in each tissue type (as shown in Figure 7D–F).

Notably, the time correlation analysis (Figure 7G) revealed that the 880 nm and 1700 nm bands exhibited the strongest correlations with the culture period, with R^2^ values of 0.50 for both bands, surpassing those of the other spectral bands. Furthermore, in terms of the response to heavy metal stress (Figure 7H), the 1670 nm band demonstrated the highest correlation with Pb concentration (R^2^ = 0.20) among all the detection bands. Although the absolute correlation values are moderate, these results are relatively significant across the tested bands, suggesting that these characteristic spectral bands could serve as potential time series monitoring indicators for plant responses to heavy metal stress.

Principal component analysis (Table 7) reveals that the response of roots to Pb stress is concentrated within the characteristic wavelength range of 1670–1675 nm, with the first two principal components (PC1 and PC2) accounting for a cumulative contribution rate of 97.50%. Under short-term stress (3 days), there was a significant increase in reflectance at 1670.28 nm, particularly in the 600 mg/L treatment group, where reflectance (0.5416) increased by 59.2% compared to CK (0.3404). However, under long-term stress (11 days), the reflectance of this band returned to baseline levels, with values comparable to CK (600 mg/L: 0.3426 vs. CK: 0.3404) (Table 8).

The first principal component (PC1) in the stem exhibited an eigenvalue of 4.9077, accounting for a contribution rate of 96.56%, which corresponds to the spectral band at 1304.31 nm (Table 9). Under short-term exposure (3 days), the reflectance at this band showed an increasing trend with elevated Pb concentration, ranging between 0.4329 (CK) and 0.5181 at 600 mg/L. In contrast, under long-term treatment (11 days), the reflectance decreased by 36.3–43.8% in the low-concentration groups (50–300 mg/L), while the high-concentration group (600 mg/L) maintained a stable reflectance value of 0.5273 (Table 10).

The spectral characteristics of the leaves exhibit a dual-band response mode (Table 11), with a synergistic effect observed between visible light at 880.85 nm (PC1, contribution rate of 75.39%) and short-wave infrared light at 1675.40 nm (PC2, contribution rate of 14.95%). Notably, under long-term high-concentration treatment (600 mg/L), the reflectance at 1675.40 nm decreased by 38.5% (0.5243) compared to the short-term peak observed at 100 mg/L (0.8526) (Table 12).

The contribution rate of PC1 in the stem (96.56%) was significantly higher than that in the root (86.91%) and leaf (75.39%), indicating that the spectral variation in the stem is highly concentrated in a single principal component. This may be attributed to its structural characteristics and Pb transport function. Both the root and leaf exhibited a response band at 1675.40 nm, suggesting that this wavelength may serve as a universal sensitive band for detecting Pb stress. Additionally, the stem-specific wavelength of 1304.31 nm may reflect its unique physiological response mechanism under stress conditions.

## 3. Materials and Methods

### 3.1. Sample Collection and Material Processing

*X. strumarium* seedlings were collected in May 2024 from the Yellow River bank in Lingbao City, Henan Province (34.6826°N, 110.9163°E), with morphologically uniform specimens (12.5 ± 0.8 cm plant height) transplanted, retaining their original rhizospheric soil, and transported to the laboratory within 4 h. Prior to hydroponic cultivation, the roots were gently rinsed with deionized water to remove the adhering soil. The hydroponic experiment included six discrete time points (1, 3, 5, 7, 9, 11 days) and five Pb^2+^ concentration levels (0, 50, 100, 300, 600 mg/L) using Pb acetate trihydrate [Pb(CH_3_COO)_2_·3H_2_O] as the Pb^2+^ source. Cultivation occurred in 2 L polypropylene containers with deionized water containing 1/4 modified Hoagland nutrient solution (pH 5.8 ± 0.2), maintaining six biological replicates per treatment (180 total seedlings) under natural indoor illumination (12 h light cycle, 22 ± 2 °C daytime temperature) with nutrient solution renewal every 3 days. Post-treatment specimens underwent triple deionized water rinsing to remove surface-adsorbed Pb, followed by shade drying and 24 h lyophilization. Two-thirds of the samples were dissected into their root–stem–leaf components, flash-frozen in liquid nitrogen, ball-milled, and sieved (120-mesh), while the remaining third were retained as whole-plant specimens. All materials were vacuum-sealed and cryopreserved at −80 °C for subsequent analysis.

### 3.2. Experimental Methods and Data Analysis

#### 3.2.1. Pb Content Detection

The relative Pb content was determined using an energy-dispersive X-ray (EDX) fluorescence spectrometer (EDX-7000, Shimadzu, Japan). Prior to analysis, the instrument was energy-calibrated with certified reference materials. Root, stem, and leaf powder samples sieved through a 120-mesh screen were uniformly dispersed in polyester film-lined containers for elemental analysis, covering a range from Al (atomic number 13) to U (atomic number 92). The absolute Pb content was analyzed using an inductively coupled plasma optical emission spectrometer (ICP-OES) (Agilent ICP-OES). For sample pre-treatment, 0.2000 g of powder was weighed into porcelain crucibles, ashed in a muffle furnace (550 °C for 300 min), cooled, mixed with 10 mL of 5% nitric acid, allowed to stand for 45 min, and filtered. The resulting solution was then subjected to ICP-OES analysis. Experimental data were statistically analyzed using one-way analysis of variance (ANOVA) in IBM SPSS Statistics software (version27), with values expressed as means of triplicate measurements. Data processing and graphical representations were performed using Microsoft Excel 2016 and Origin 2024b.

#### 3.2.2. Fourier Transform Infrared Spectroscopy (FTIR) Study

The chemical group characterization of the root, stem, and leaf powders treated at 3, 7, and 11 days was performed using FTIR (Nicolet iS10, Thermo Fisher Scientific, Billerica, MA, USA). The powders were prepared via the potassium bromide (KBr) pellet method. The spectral parameters were set to a wavenumber range of 4000–400 cm^−1^, a resolution of 4 cm^−1^, and 32 scanning accumulations. Background subtraction was conducted using a pure KBr blank reference prior to measurement. Raw spectra were baseline-corrected and smoothed using OMNIC software, then exported to Origin 2024b for spectral plotting.

#### 3.2.3. Biomass Pyrolysis Analysis

The thermal decomposition characteristics of the root, stem, and leaf tissues were analyzed using thermogravimetric-differential scanning calorimetry (STA8000, PerkinElmer, Worcester, MA, USA). Control (CK), root, stem, and leaf samples subjected to 50 mg/L Pb^2+^ for 3 days, 50 mg/L Pb^2+^ for 9 days, and 600 mg/L Pb^2+^ for 9 days were selected for analysis. The powder samples (10.00 ± 0.05 mg) were placed in alumina crucibles and analyzed under a nitrogen atmosphere (flow rate: 20 mL/min). The temperature program was set to equilibrate at 30 °C for 2 min, followed by heating to 800 °C at 10 °C/min, with a final hold at 800 °C for 5 min. The raw data were first processed using Microsoft Excel 2016, and thermogravimetric analysis (TGA) and derivative thermogravimetry (DTG) curves were subsequently generated with Origin 2024b for analytical purposes.

#### 3.2.4. Hyperspectral Analysis

Plant spectral characteristics were analyzed using a hyperspectral imaging system (HIS-NIR-640, Wuling Optics, Taiwan) comprising a lens assembly, lighting, motorized stage, and dark chamber. Whole *X. strumarium* specimens subjected to 3-day and 11-day Pb^2+^ exposures (0, 50, 100, 300, 600 mg/L) were positioned on black background plates within the light-sealed chamber. Lens calibration was performed prior to spectral acquisition across 850–1750 nm wavelengths using the manufacturer’s Spectral Image NIR software. Regions of interest (ROIs) encompassing root, stem, and leaf tissues were manually delineated to extract full-band average reflectance values. Acquired spectral data underwent statistical correlation analysis and principal component analysis using Microsoft Excel 2016 and Origin 2024b for processing and visualization.

## 4. Discussion

### 4.1. Organ-Specific Accumulation and Defense Mechanisms

EDX and ICP-EOS analyses revealed distinct tissue-specific accumulation patterns of Pb in plants, with all the peak accumulations occurring exclusively under the 600 mg/L treatment. The root tissues exhibited the highest relative Pb content (66.56%) at 9 days, while the stems (1.20%) and leaves (0.16%) reached their peak levels at 5 days. Absolute quantification by ICP-EOS confirmed the maximal Pb accumulation in roots (56,328.21 mg/kg at 9 days), exceeding the stem (7036.34 mg/kg) and leaf (233.82 mg/kg) concentrations measured at their 5-day peaks. Root systems retained 88.55% of the total absorbed Pb, with the stems and leaves contributing only 11.14% and 0.31%, respectively. These results demonstrate preferential Pb sequestration in root tissues. The observed distribution pattern of root interception, stem blockage, and leaf exclusion aligns with the defense strategies of various heavy metal hyperaccumulating plants [38]. This mechanism likely restricts the migration of Pb^2+^ to photosynthetic organs through processes such as cell wall binding, vacuolar compartmentalization, and chelating protein-mediated immobilization [39,40].

The stem Pb^2+^ accumulation in the 600 mg/L treatment group displayed time-dependent enhancement, peaking 4–6 days later than in the roots. This temporal shift implies the potential activation of vascular active transport systems (potentially involving HMA transporters [41]) under elevated Pb^2+^ exposure, though tightly regulated to preserve aerial organ functionality. A nonlinear escalation in Pb^2+^ accumulation occurred above 300 mg/L, with levels surpassing 45%, demonstrating a critical concentration threshold for Pb absorption in *X. strumarium*. Short-term exposure (1–3 days) elicited rapid root accumulation (13.62%/d at 600 mg/L), indicating the dominance of extracellular vesicle-mediated transport or ion channel pathways [42]. Prolonged stress (11 days) reduced root accumulation by 21.8%, which can be potentially attributed to compromised membrane integrity facilitating Pb^2+^ efflux or impaired antioxidant capacity disrupting ion homeostasis [43,44]. While this bi-phasic pattern parallels cadmium accumulation dynamics in rice roots [45], *X. strumarium* exhibited superior Pb^2+^ tolerance, highlighting species-specific detoxification adaptations.

The phytotoxic manifestations of elevated Pb^2+^ exposure (≥300 mg/L), particularly root biomass reduction and chlorosis, align with lead’s established interference in mitotic processes, nutrient assimilation impairment, and oxidative stress generation [46,47]. Notably, stem necrosis exhibited a temporal correlation with peak stem Pb^2+^ accumulation (days 5–9), strongly implicating xylem dysfunction or vascular tissue collapse as primary drivers of aerial organ mortality [48].

*X. strumarium* demonstrates notable Pb^2+^ resilience through tissue-specific compartmentalization and spatiotemporal accumulation control, with exceptional root enrichment capacity positioning it as a promising phytoremediation candidate. Nevertheless, membrane peroxidation and metabolic pathway disruption under high Pb^2+^ stress remain critical implementation barriers. Strategic optimization through multi-omics-guided pathway engineering and rhizosphere microbiome manipulation could enhance remediation efficiency while maintaining ecological safety thresholds.

### 4.2. Dynamic Response of Functional Groups Regulating Pb Chemical Immobilization

Lead-induced molecular architecture reorganization in plants was quantitatively monitored through FTIR by tracking time-dependent peak variations. Comparative functional group analysis across *X. strumarium* root, stem, and leaf tissues under Pb^2+^ exposure uncovered a multilayer defense strategy against metal toxicity, involving the sequential activation of cell wall polysaccharide modification, secondary metabolite complexation, and organelle-specific sequestration pathways.

Under acute Pb^2+^ stress (3 days, 600 mg/L), the roots exhibited an upshift in the 1380–1420 cm^−1^ region (1419 cm^−1^ vs. CK 1385 cm^−1^), signifying pectin carboxyl (COO^−^) de-esterification to enhance Pb^2+^ chelation via exposed free carboxyl groups [49]. This trend stabilized at 1384–1387 cm^−1^ by Day 7, suggesting the saturation of pectin–Pb complexation. The concurrent enhancement at 778 cm^−1^ implicated phosphate groups (e.g., cell wall-bound phytic acid/phospholipids) in Pb^2+^ coordination through P-O bonds [50]. Prolonged exposure (11 days, 600 mg/L) reduced root amide I band intensity (1630–1640 cm^−1^ to 1607 cm^−1^), indicative of ROS-induced protein conformational changes or degradation [51]. Low–medium concentrations (50–100 mg/L) maintained spectral stability (Δν < 5 cm^−1^), confirming efficient vacuolar Pb^2+^ sequestration via ABC transporters [52]. In stems under chronic stress, spectral convergence at 1382–1386 cm^−1^ with stable intensity, coupled with dominant phytic acid signatures at 777 cm^−1^, suggested vascular Pb^2+^ chelation via secreted inositol hexaphosphate to mitigate vascular blockage [53,54]. Leaves at 100 mg/L showed amplified 1632–1652 cm^−1^ bands (phenolics/lignin), reflecting oxidative polymerization to create Pb^2+^ diffusion barriers in mesophyll [55].

The lipid metabolism-associated band at 2925 cm^−1^ (C-H stretch) remained stable across tissues (Δν < 3 cm^−1^), confirming preserved membrane phospholipid integrity [56]. Stem O-H vibrations at 3418 cm^−1^ (vs. 3390–3400 cm^−1^ baseline) under chronic stress implied osmotic adjustment via hydrophilic metabolite accumulation (e.g., soluble sugars) [57]. Leaves under acute stress displayed elevated 3406 cm^−1^ O-H signals alongside attenuated cuticular C-O-C vibrations (1030 cm^−1^), revealing a dual adaptation: cuticle thickening for Pb^2+^ exclusion coupled with intracellular hydrophilic solute accumulation for water homeostasis [58].

Root systems primarily immobilize Pb^2+^ through pectin carboxyl coordination and lignin–Pb complexation, whereas stems employ organic-acid-mediated soluble chelation to mitigate vascular toxicity. Foliar tissues restrict Pb^2+^ influx via phenolic polymerization barriers and cuticular structural reinforcement. Nevertheless, two critical limitations persist: (1) high-concentration-induced protein conformational instability (evidenced by root amide I band redshift to 1607 cm^−1^) and (2) saturation thresholds in vacuolar sequestration capacity (reflected in leaf 533 cm^−1^ peak stabilization), both constraining phytoremediation efficiency. Field validation must address soil–plant feedback effects on infrared spectral dynamics [39,40,41,42,43,44,45,46,47,48], particularly rhizosphere organic matter interference and root exudate-mediated peak variations, to improve diagnostic reliability in heterogeneous contaminated matrices.

### 4.3. Evolution of Thermal Stability Under Lead-Induced Stress

Pb stress impacts plant systems through multidimensional metabolic reprogramming, extending beyond observable morphological alterations and ionic distribution patterns to encompass the structural reconfiguration of cellular constituents and adaptive responses. This investigation systematically deciphers Pb^2+^-induced thermal decomposition signatures in root, stem, and leaf tissues via synchronized TGA and DTG. By correlating pyrolysis curves with exposure time and concentration gradients, the regulatory mechanisms of Pb stress on plant cellular structures and metabolites can be revealed.

Low-temperature dehydration (30–150 °C): The DTG peak intensification at 65 °C (28% increase vs. CK) and reduced residual mass at 129 °C in Pb^2+^-stressed roots indicate disrupted water compartmentalization, likely from membrane-permeability alterations accelerating free/loosely bound water evaporation [59]. The stems exhibited concentration-dependent weight loss reductions (42% decrease at 9d/600 mg/L vs. CK), suggesting cell wall reinforcement via polysaccharide cross-linking (e.g., hemicellulose networking) rather than passive hydrophobe accumulation [60,61]. Leaf dehydration resistance (57.2% lower weight loss at 9d/600 mg/L) correlated with cuticular thickening, a dual adaptive strategy to limit stomatal Pb^2+^ uptake and reduce transpirational water loss [62].

Medium-temperature pyrolysis (200–400 °C): Root hemicellulose/pectin decomposition (288 °C peak attenuation in Pb^2+^ groups) revealed metal-induced cell wall remodeling, where pectin–Pb crosslinking and lignin deposition suppressed polysaccharide degradation while enhancing metal immobilization [63,64]. The stem thermograms showed suppressed exothermic peaks at 280–350 °C (minimal intensity at 9d/600 mg/L), evidencing Pb^2+^-impaired cellulose biosynthesis or microfibril disorientation, phenotypically manifested as vascular wilt. Conversely, leaf DTG peak intensification (0.51%/ °C at 9d/600 mg/L) with delayed cumulative decomposition suggests lignin condensation and cellulose microfibril densification to preserve structural integrity under stress.

High-temperature carbonization (450–800 °C): Elevated root residual carbon (66.7% increase at 9d/600 mg/L vs. CK) aligned with lignin–Pb complex accumulation, which stabilizes organic matrices against thermal volatilization [65]. The stem/leaf residual carbon increments (e.g., 33.6% stem increase at 9d/600 mg/L) reinforced this mechanism. However, the near-CK residual levels in leaves under prolonged low-dose stress (9d/50 mg/L) implied preferential vacuolar Pb^2+^ partitioning over cell wall binding, highlighting dose-dependent subcellular sequestration strategies [66].

Thermogravimetric profiling revealed distinct Pb^2+^ dose–time duality across the organs. The roots exhibited a 130% intensification of the 420–460 °C secondary decomposition peak in the 9d/600 mg/L group versus CK, while the 9d/50 mg/L group showed a 19.4% peak reduction compared to the 3d/50 mg/L group. This dichotomy suggests that chronic high-Pb^2+^ exposure drives progressive cell wall lignocellulose restructuring [67,68], whereas low-dose conditions enable the partial recovery of catabolic activity through redox-regulated decomposition pathways [69]. Stem thermograms further confirmed temporal adaptation trajectories, with 22% greater low-temperature weight loss in the 3d/50 mg/L group versus the 9d/50 mg/L group, demonstrating hydraulic acclimatization to sustained low-level Pb^2+^ stress.

Pb stress induces root pectin de-esterification (FTIR-confirmed) and lignin deposition (residual carbon rate increase), enhancing Pb^2+^ cell wall fixation while suppressing hemicellulose degradation (medium-temperature peak intensity reduction). The stems reduce water diffusion rates via cell wall polysaccharide network adjustments (low-temperature weight loss decrease), whereas the leaves alleviate oxidative damage through lignin condensation (high-temperature residual carbon increase), synergizing with antioxidant systems. These findings demonstrate *X. strumarium*’s root-specific Pb^2+^ immobilization efficiency and stem–leaf adaptive plasticity, highlighting its applicability in moderately Pb^2+^-polluted environments.

### 4.4. Spectral Fingerprints of Lead Stress Evolution

Plant spectral reflectance patterns can be directly determined by physiological–biochemical traits undergoing stress-induced modifications, with near-infrared (NIR) signatures (750–2500 nm) proving particularly diagnostic for growth monitoring. This spectral window captures the characteristic absorption bands of C-H, N-H, and O-H molecular bonds through their vibrational overtones [70], enabling hyperspectral imaging to simultaneously resolve cellular structural changes (700–1300 nm) and hydration dynamics (1300–2500 nm) [71,72].

The significant response of the roots in the 1670–1675 nm wavelength range (PC1 contribution rate 86.91%) and their increased reflectance under short-term stress (0.3404 to 0.5416) reflect the close correlation between cell wall reconstruction (proteins, lignin) and starch components [73]. Under long-term stress (11 days), the decreased reflectance may result from excessive cell wall cross-linking (increased structural fragility) or starch-hydrolysis-induced cellular collapse [74], consistent with FTIR observations (amide I band redshift) and thermogravimetric data (lignin–Pb saturation). The stem’s high PC1 contribution rate (96.56%) at 1304.31 nm indicates its near-infrared sensitivity, likely associated with vascular tissue structural changes and water status variations [75]. Short-term stress caused fluctuating reflectance increases with concentration (CK: 0.4329; 600 mg/L: 0.5181), while long-term low-concentration exposure (11 days, 50 mg/L) sharply reduced reflectance (0.2430), suggesting that stems adapt via short-term structural modifications (e.g., lignin deposition) but suffer suppressed physiological activity under chronic low-dose stress. The leaf dual-band responses at 880.85 nm and 1675.40 nm (cumulative contribution rate 90.34%) reflect coordinated changes in photosynthetic pigments (e.g., chlorophyll) and cell wall components [76,77]. The short-term high-concentration reflectance increase (3 days, 300 mg/L: 0.8293) may arise from stratum corneum thickening or oxidative product accumulation, whereas the long-term high-concentration decrease (11 days, 600 mg/L: 0.5243) likely indicates photosystem II damage or chloroplast structural disintegration [78,79]. Under short-term high-concentration stress (3d, ≥300 mg/L), universal organ reflectance increases may result from membrane lipid peroxidation or cell wall rupture-induced internal structure loosening [80]. Long-term stress (11 days) induced differentiated responses: the low-concentration groups (50–100 mg/L) showed reduced root–stem reflectance (e.g., stem 1304.31 nm: 0.2430), possibly reflecting metabolic resource allocation to repair processes (e.g., antioxidant enzyme synthesis) that reduce light scattering loss [75], indicating plant self-repair mechanisms. The high-concentration root reflectance decrease suggests irreversible damage, while the stem reflectance increase (0.5273) may derive from vascular tissue secondary wall thickening or enhanced vacuolar segregation.

These hyperspectral signatures reveal that acute Pb^2+^ stress primarily activates plant structural adaptations (cell wall remodeling/keratinization thickening), with reflectance increases serving as early stress biomarkers. Chronic exposure induces reflectance divergence—low-concentration decline versus high-concentration rebound—mirroring the metabolic trade-off between damage repair and resource reallocation. Future work should prioritize developing inversion models targeting three diagnostic bands: 880.85 nm (leaf phenolic barriers), 1304.31 nm (stem hydration states), and 1675 nm (root cell wall restructuring), thereby establishing a spectral fingerprint database for field-deployable, nondestructive phytoremediation monitoring.

### 4.5. Study Limitations and Future Research

This study employed Pb(II) acetate trihydrate in hydroponic systems to simulate Pb stress, a soluble form that exhibits superior bioavailability compared to natural soil-bound Pb species (e.g., PbCO_3_, PbSO_4_) [81]. While hydroponic systems effectively elucidate plant Pb^2+^ tolerance mechanisms, these findings may differ from field conditions where Pb speciation, organic matter complexation, and rhizospheric microbial interactions critically influence plant uptake and immobilization efficiency. Future investigations should validate the identified critical concentration threshold (300 mg/L) and spectroscopic biomarkers through in situ soil experiments under environmentally relevant Pb exposure scenarios.

Building upon established research documenting Pb-induced antioxidant system alterations (e.g., SOD activity, MDA accumulation) [82,83,84], our spectral and thermogravimetric analyses revealed dynamic cell wall remodeling processes—including pectin demethylation and lignin–Pb complex formation—thereby complementing conventional biochemical assays with molecular-level structural insights. Subsequent research will be expanded to include the following: (1) the systematic quantification of *X. strumarium*’s classical physiological indices under Pb stress; (2) integrated transcriptomic analyses to verify key gene expression patterns in pectin/lignin biosynthesis pathways, thereby refining the molecular mechanism framework.

## 5. Conclusions

*X. strumarium* orchestrates Pb distribution through tripartite compartmentalization—root interception (peaking at 56,328.21 mg/kg, 88.55% total accumulation), stem blockage (7036.34mg/kg, 11.14%), and leaf exclusion (233.82mg/kg, 0.31%)—demonstrating a universal phytostabilization strategy via spatial metal partitioning. This study pioneers the identification of Pb^2+^ absorption threshold behavior (nonlinear accumulation surge >300 mg/L) and mechanistically confirms that root immobilization relies on cell wall pectin de-esterification (COO^−^ exposure) coupled with lignin–Pb complexation.

This work innovatively integrates EDX, ICP-OES, FTIR, thermogravimetric, and hyperspectral analyses to decode spatiotemporal linkages between Pb^2^-induced plant component reorganization (e.g., carboxyl exposure, lignin deposition) and spectral signatures (1670 nm band sensitivity). While *X. strumarium* demonstrates superior biomass production and ecological adaptability, its stem–leaf Pb^2+^ accumulation limitations position it as an optimal candidate for in situ rhizospheric Pb^2+^ immobilization rather than phytoextraction. We further validated Pb^2+^’s dual transport modes: passive apoplastic migration through root extracellular matrices and stress-activated vascular translocation (4–6 day stem accumulation delay). The analysis of these multidimensional response mechanisms establishes a scientific foundation for the screening and engineering of heavy metal-tolerant plants.

## Figures and Tables

**Table 1:** Main infrared bands and characteristics of roots.

Infrared Band	Main Characteristics
3400–3390 cm^−1^	At Day 3, the 600 mg/L group’s wavenumber dropped from 3416 to 3391 cm^−1^ (CK). At Day 7 and Day 11, all groups’ wavenumbers stabilized near 3395 cm^−1^.
2925 cm^−1^	The C-H wavenumbers were stable within 2922–2928 cm^−1^ across all concentrations and time points.
1630–1640 cm^−1^	The 600 mg/L group dropped significantly to 1607 cm^−1^ on Day 11 (1631 cm^−1^ on Day 3).
1380–1420 cm^−1^	After Day 3 at 600 mg/L, the COO^−^ wavenumber rose to 1419 cm^−1^ and then stabilized at 1384–1387 cm^−1^ with prolonged stress (Day 7–11).
500–600 cm^−1^	At Day 3, the 100 mg/L group showed a disturbance at 778 cm^−1^. At Day 11, the 600 mg/L group showed on at 777 cm^−1^.

**Table 2:** Main infrared bands and characteristics of stems.

Infrared Band	Main Characteristics
3400–3390 cm^−1^	After Day 3, the 600 mg/L Pb^2+^ group increased its O-H wavenumber from 3384 cm^−1^ (CK) to 3390 cm^−1^. After Day 11, all groups’ wavenumbers neared 3418 cm^−1^.
2916–2924 cm^−1^	The C-H wavenumbers of all the treated groups were stable within 2916–2924 cm^−1^.
1621–1632 cm^−1^	High-concentration (600 mg/L): Stabilized at 1632 cm^−1^ on Day 11. Low-concentration (100–300 mg/L): Fluctuated at 1622–1630 cm^−1^. Long-term (Day 11): All groups stabilized at 1382–1386 cm^−1^.
1382–1417 cm^−1^	Short-term (Day 3): The high-concentration (600 mg/L) COO^−^ wavenumber was 1417 cm^−1^, the same as for CK. The 300 mg/L group’s wavenumber decreased to 1386 cm^−1^. All the groups stabilized at 1382–1386 cm^−1^.
500–620 cm^−1^	At Day 11, the 100 mg/L group showed a peak at 777 cm^−1^, while the high-concentration group (600 mg/L) only had a peak at 537 cm^−1^.

**Table 3:** Main infrared bands and characteristics of leaves.

Infrared Band	Main Characteristics
3370–3406 cm^−1^	After Day 3, the high-concentration (600 mg/L) O–H wavenumber rose significantly to 3406 cm^−1^ from CK’s 3396 cm^−1^. After Day 11, all groups’ wavenumbers converged to 3376–3399 cm^−1^.
2919–2924 cm^−1^	The C-H wavenumbers of all the treated groups were stable within 2919–2924 cm^−1^.
1632–1652 cm^−1^	(100 mg/L): The wavenumber rose to 1652 cm^−1^ on Day 3 (CK: 1646 cm^−1^). (600 mg/L): The wavenumber dropped to 1641 cm^−1^ on Day 11 (1632 cm^−1^ on Day 3).
1384–1439 cm^−1^	After Day 11, all groups’ wavenumbers stabilized at 1384–1416 cm^−1^.
1030–1106 cm^−1^	At Day 7, the 600 mg/L group’s wavenumber dropped to 1065 cm^−1^.
500–620 cm^−1^	Low-concentration group (100 mg/L): Detected a vibration at 526 cm^−1^ on Day 3, dropping to 522 cm^−1^ on Day 11.

**Table 4:** Main peak changes and residual carbon rates in thermal analysis curves of *X. strumarium* roots under different treatments.

Treatment Group	Temperature Stage (°C)	TGA Residual Amount (%)	DTG Peak (mg/min)	Residual Carbon Rate at 800 °C (%)
CK	287–394	69.3 to 48.8	−0.385 (287 °C)	19.1
3d 50 mg/L Pb	284–384	69.9 to 52.0	−0.300 (284 °C)	25.2
9d 50 mg/L Pb	287–402	69.2 to 48.5	−0.372 (287 °C)	19.8
9d 600 mg/L Pb	292–387	74.4 to 56.1	−0.328 (292 °C)	31.8

**Table 5:** Main peak changes and residual carbon rates in thermal analysis curves of *X. strumarium* stems under different treatments.

Treatment Group	Temperature Stage (°C)	TGA Residual Amount (%)	DTG Peak (mg/min)	Residual Carbon Rate at 800 °C (%)
CK	289–370	63.2 to 45.5	−0.400 (289 °C)	17.7
3d 50 mg/L Pb	292–382	69.9 to 49.9	−0.382 (293 °C)	24.4
9d 50 mg/L Pb	290–382	66.0 to 47.8	−0.401 (290 °C)	20.8
9d 600 mg/L Pb	292–373	69.2 to 51.2	−0.401 (292 °C)	23.7

**Table 6:** Main peak changes and residual carbon rates in thermal analysis curves of *X. strumarium* leaves under different treatments.

Treatment Group	Temperature Stage (°C)	TGA Residual Amount (%)	DTG Peak (mg/min)	Residual Carbon Rate at 800 °C (%)
CK	289–390	68.8 to 52.5	−0.242 (290 °C)	22.7
3d 50 mg/L Pb	290–400	74.2 to 56.9	−0.273 (29 °C)	28.6
9d 50 mg/L Pb	288–395	71.3 to 52.1	−0.317 (289 °C)	21.5
9d 600 mg/L Pb	290–396	73.2 to 54.4	−0.310 (291 °C)	24.8

**Table 7:** Principal component analysis statistical results of roots.

Principal Component	Eigenvalue	Contribution Rate (%)	Cumulative Contribution Rate (%)	Maximum Contribution Wavelength (nm)
1	0.6641	86.91	86.91	1670.28
2	0.0809	10.59	97.50	1675.40
3	0.0163	2.13	99.63	889.32
4	0.0020	0.26	99.89	1728.40
5	0.0005	0.07	99.96	1731.83
6	0.0002	0.03	99.99	1671.99

**Table 8:** Reflectance values at maximum contribution wavelengths of roots under different conditions.

Maximum Contribution Wavelength (nm)	CK	3d 50 mg	3d 100 mg	3d 300 mg	3d 600 mg	11d 50 mg	11d 100 mg	11d 300 mg	11d 600 mg
1670.28	0.3404	0.4044	0.3745	0.4482	0.5416	0.3208	0.4111	0.4517	0.3426
1675.40	0.3430	0.4077	0.3797	0.4675	0.5464	0.3322	0.4241	0.4667	0.3372
889.32	0.2051	0.1827	0.1932	0.2174	0.2269	0.1617	0.1938	0.1752	0.1937
1728.40	0.2894	0.3448	0.3197	0.3688	0.3811	0.299	0.3331	0.371	0.2483
1731.83	0.2871	0.3321	0.3094	0.364	0.3832	0.2976	0.3266	0.3555	0.2555
1671.99	0.3431	0.4072	0.3798	0.4607	0.5496	0.3281	0.4201	0.4602	0.3426

**Table 9:** Principal component analysis statistical results of stems.

Principal Component	Eigenvalue	Contribution Rate (%)	Cumulative Contribution Rate (%)	Maximum Contribution Wavelength (nm)
1	4.9077	96.56	96.56	1304.31
2	0.1120	2.20	98.77	1675.40
3	0.0576	1.13	99.90	1738.69
4	0.0038	0.07	99.98	1735.26
5	0.0006	0.01	99.99	885.93
6	0.0003	0.01	99.99	1719.83

**Table 10:** Reflectance values at maximum contribution wavelengths of stems under different conditions.

Maximum Contribution Wavelength (nm)	CK	3d 50 mg	3d 100 mg	3d 300 mg	3d 600 mg	11d 50 mg	11d 100 mg	11d 300 mg	11d 600 mg
1304.31	0.4329	0.4754	0.4971	0.4794	0.5181	0.2430	0.1861	0.3265	0.5273
1675.40	0.4951	0.5793	0.5858	0.7398	0.5962	0.3639	0.2982	0.4878	0.4779
1738.69	0.3685	0.4217	0.3833	0.4189	0.4298	0.2809	0.2463	0.3443	0.3275
1735.26	0.3759	0.4258	0.3757	0.4249	0.4271	0.2771	0.255	0.3405	0.3482
885.93	0.2825	0.2858	0.3009	0.2762	0.3392	0.1827	0.1526	0.2129	0.2807
1719.83	0.3738	0.4456	0.4132	0.4643	0.4495	0.3050	0.2726	0.3621	0.3392

**Table 11:** Principal component analysis statistical results of leaves.

Principal Component	Eigenvalue	Contribution Rate (%)	Cumulative Contribution Rate (%)	Maximum Contribution Wavelength (nm)
1	0.8526	75.39	75.39	880.85
2	0.1691	14.95	90.34	1675.40
3	0.1030	9.11	99.45	1675.40
4	0.0054	0.48	99.92	1735.26
5	0.0004	0.04	99.96	1663.46
6	0.0003	0.03	99.99	1661.76

**Table 12:** Reflectance values at maximum contribution wavelengths of leaves under different conditions.

Maximum Contribution Wavelength (nm)	CK	3d 50 mg	3d 100 mg	3d 300 mg	3d 600 mg	11d 50 mg	11d 100 mg	11d 300 mg	11d 600 mg
880.85	0.4462	0.3786	0.4413	0.4086	0.4416	0.3325	0.2911	0.2771	0.2168
1675.40	0.6869	0.7220	0.7526	0.8293	0.8019	0.7172	0.8526	0.7894	0.5243
1675.40	0.6869	0.7220	0.7526	0.8293	0.8019	0.7172	0.8526	0.7894	0.5243
1735.26	0.4798	0.5034	0.5433	0.5628	0.5071	0.4704	0.4755	0.4176	0.2807
1663.46	0.6291	0.6419	0.6753	0.6749	0.6830	0.6122	0.7205	0.6398	0.5218
1661.76	0.6054	0.6136	0.6477	0.6370	0.6487	0.5821	0.6835	0.6025	0.5136

## Data Availability

The original contributions presented in this study are included in the article. Further inquiries can be directed to the corresponding author.
